# Clinician Perspectives on mRehab Interventions and Technologies for People with Disabilities in the United States: A National Survey

**DOI:** 10.3390/ijerph16214220

**Published:** 2019-10-31

**Authors:** John Morris, Mike Jones, Nicole Thompson, Tracey Wallace, Frank DeRuyter

**Affiliations:** 1 Shepherd Center, Atlanta, GA 30309, USA; mike.jones@shepherd.org (M.J.); nicole.thompson@shepherd.org (N.T.); tracey.wallace@shepherd.org (T.W.); 2 Department of Surgery, Duke University Medical Center, Durham, NC 27708, USA; frank.deruyter@duke.edu

**Keywords:** mobile health, mHealth, mRehab, disability, rehabilitation, information and communication technology

## Abstract

Mobile health and mobile rehabilitation (mHealth and mRehab) services and technologies have attracted considerable interest from healthcare providers, technology vendors, rehabilitation engineers, investors and policy makers in recent years. Successful adoption and use of mHealth/mRehab requires clinician support and engagement, including the ability to identify appropriate use cases and possible barriers to use for themselves and their patients, and acquire adequate knowledge and confidence using mHealth/mRehab interventions. This article reports results from a survey of rehabilitation clinicians in the United States on their attitudes, experience, expectations and concerns regarding mHealth/mRehab interventions and technologies. Over 500 clinicians in physical, occupational, speech, recreation and psychological therapy professions, among others, participated in the survey. Respondents reported that an overwhelming majority of their patients need additional therapy after discharge from inpatient environments, and over half of outpatients need additional therapy between visits. A large majority reported prescribing specific exercises and interventions for patients to work on outside of the clinic. However, only 51% reported being comfortable integrating mRehab technology into their practice; and only 23% feel knowledgeable about rehabilitation technology currently available. Technologies to support mRehab are maturing rapidly. Clinicians recognize the need for mRehab, but their knowledge and confidence prescribing mRehab represents a significant barrier to adoption.

## 1. Introduction

Technologies that have transformed education, entertainment and business are now reaching the level of maturity needed to support robust, remote mobile healthcare. As we leverage technology in all facets of our lives, we are now witnessing how the utilization of smart phones, wearable devices, apps and sensors, smart home hubs for automation/control, environmental sensors, cloud computing, and high-speed networks are vastly expanding the foundation for effective mobile care. The term mobile health (mHealth), is widely understood to refer to the delivery of health care services via mobile communication devices. The Research Plan on Rehabilitation published by the United States National Institute of Health notes that “…mHealth is becoming a significant part of the healthcare … economy. … the use of ICT [Information and Communication Technology] can broaden rehabilitation and healthcare research opportunities for researchers and service opportunities for patients” [1].

A more recent term is mobile rehabilitation (mRehab), which refers to the delivery of rehabilitation services using mobile ICT [2]. The current state and future needs of mobile healthcare for people with disabilities have recently been clearly identified [3]. Examples of emerging work in this area is reflected in recent publications that include a review of rehabilitation mobile health apps [4,5], use of technology in a home-based stroke rehab program [6] and use of mHealth technologies by people with vision impairment [7]. Further, the United States National Institutes of Health (NIH) Research Plan recognizes the potential of mRehab applications including “…symptom monitoring, real-time data capture, real-time access to information about (patients) navigating the community, social connectedness through peer-to-peer support, and bidirectional communication” [1]. Finally, it is imperative to recognize that digital healthcare can involve different levels of remote management, from full (by health care professions) to self-management (by patients).

Although potentially game-changing in providing greater access to affordable rehabilitation services, digital health applications for both mHealth and mRehab remain limited by narrow functionality [8], uncertain measurement accuracy of sensors [9,10], uneven durability and usability, and high rates of user abandonment [11]. Furthermore, with the breakneck speed that digital health is evolving, recent concerns have been raised about underserved patient groups communities being left behind [12]. Even if these limitations are overcome, issues of acceptance by patients and providers must also be recognized and addressed. Issues of acceptance by consumers include familiarity and comfort with use of technology, human-technology interface limitations for people with disabilities, concerns about privacy and intrusiveness, and loss or diminished contact with the clinical service provider (less personalized care). Potential benefits include greater ease of access to services, greater empowerment for one’s own health outcomes and the opportunity to progress in therapy at the desired pace. 

Potential barriers to acceptance of mRehab by clinicians include lack of familiarity with technology, complexity and time requirements for use of the technology, practice needed to become proficient, changes required in clinical workflow to incorporate the approach, and the need for practice standards and evidence of effectiveness to govern and justify use of the approach [13,14,15].

To gain a better understanding of potential barriers and facilitators that may influence adoption of mRehab tools and strategies from the clinical perspective, we conducted a survey of professionals in physical medicine and rehabilitation fields. This article presents findings from this preliminary survey and suggest directions for future work to address barriers identified by survey respondents.

## 2. Materials and Methods 

With input from our clinician advisors and informed by a review of the available literature on barriers to adoption of digital health interventions, we developed and refined the mRehab survey questionnaire (see Supplementary Materials), consisting of 22 questions, to address four broad topics: 1) perceived need for mRehab interventions based on patients’ therapy needs post-discharge from inpatient rehabilitation or between outpatient clinic visits; 2) opinions about the potential utility of mRehab interventions including the most important use cases for the technology, 3) perceived barriers to use of mRehab, including personal interest or reservations about mRehab interventions; and 4) current interest in, knowledge about, or actual experience with use of mRehab strategies. The survey also included a short section on perceptions and knowledge of cloud-based coaching/therapy platforms that can integrate and deliver multiple interventions and capture patient-generated data from multiple devices and other sources.

Participants were recruited through the researchers’ personal networks at Shepherd Center, Duke University Medical Center, the American Congress of Rehabilitation Medicine, American Physical Therapy Association, American Occupational Therapy Association, American Speech-Hearing Association, and others. Data were collected in January and February 2019 using convenience sampling methods and online data collection on the Survey Monkey web-based platform. Although no protected health information (PHI) was collected in this survey, the Survey Monkey platform does meet the privacy and security requirements of the United States Health Insurance Portability and Accountability Act of 1996 (HIPAA), which establishes essential policies and practices for protecting patient health information from unnecessary and unauthorized access. 

Efforts were made to ensure that relative balance in the number of respondents among the 4 core clinical therapy professions (physical, occupational, speech therapy and recreation therapy) by creating unique “collectors” in Survey Monkey and setting limits on the number of respondents to each. A small incentive—a $5.00 Starbucks gift card sent electronically—was offered to respondents to encourage higher levels of completeness in survey responses. The Research Review Committee at Shepherd Center reviewed and approved this research to ensure protection of participants.

## 3. Results

Response data were analyzed using SPSS version 22 (IBM, Armonk, NY, USA). A total of 505 rehabilitation clinicians across multiple specialties, including physical, occupational, speech therapy and recreation therapy, as well as psychology and other professions, completed the questionnaire (Table 1). About half of respondents reported between five and 19 years of experience in their profession, and slightly more than half (55%) personally owned a wearable fitness tracker, smart watch or other wearable device with sensors. Table 1 below provides a summary of survey respondents by profession. The “Other” category includes physical therapy assistant, certified occupational therapy assistant (COTA), medical assistant, rehabilitation instructor, experimental psychologist, and other professions.

Many respondents reported treating multiple patient populations including those with acquired brain injury (ABI), neurodegenerative diseases (NDD), musculoskeletal injury/disorder, cardiovascular disease (CVD), cancer, spinal cord injury (SCI) and other conditions (Table 2).

Similarly, many respondents reported working in multiple clinical environments, including inpatient and outpatient environments, as well as skilled nursing facility, home health and other environments (Table 3).

Respondents reported that more than 70% of their patients need additional therapy after discharge and more than 55% need additional therapy between visits to outpatient programs (Table 4). The medical specialties (physician, physician assistant, nurse and nurse practitioner) reported lowest percentages for patients needing additional therapy post-discharge and between outpatient visits. Mental health professionals (83%) and recreation therapists (80%) reported the highest percentages of patients needing additional intervention post-discharge. For outpatients, recreation therapists reported the highest percentages of patients needing additional therapy between visits.

Almost all clinicians recognized the potential of mRehab—over 95% indicated mRehab interventions could be effective in supporting post-acute and between-visits therapy interventions for their patients. Over 70% of respondents reported prescribing specific exercises and interventions to their patients to work on outside of the clinic (Table 5).

Despite the perceived need for mRehab interventions, only about half of respondents (51%) reported being comfortable integrating mRehab into their practice and less than a quarter (23%) believe they are knowledgeable about rehabilitation technologies that could be used in their clinical specialty or for their patient populations (Table 6).

The age of respondents was examined as a possible determinant of comfort integrating mRehab into practice and perceived knowledge of available mRehab solutions. Older respondents might be expected to be less comfortable integrating mRehab into practice and less knowledgeable about available technologies. Table 7 and Table 8 show the crosstabulations for respondent age (consolidated into four age ranges) with comfort and knowledge levels. 

Age was shown not to be a determinant of comfort integrating mRehab into practice or knowledge of mRehab technology. Crosstab analysis using gamma, resulted in very weak relationships between age and comfort, and age and knowledge (gamma coefficients of 0.016 and 0.036) with insignificant p-values (0.750 and 0.552, respectively).

Length of professional service was also examined as a possible determinant of a comfort integrating mRehab into practice and perceived knowledge of available mRehab solutions. Longer time in the profession might cause a clinician to be less likely to deviate from established practice. Crosstab analysis produced gamma coefficients of the strength of the relationship between length of professional service and comfort integrating mRehab into practice (0.051) and perceived knowledge (0.022), with insignificant p-values (0.317 and 0.651, respectively). 

Respondents were asked to identify the top three barriers to adoption of mRehab technology into practice. Table 9 lists the percentage of participants who selected each of the eight potential barriers listed in the questionnaire. Respondents had the option to add “other” barriers; and 35 other responses were added. Additional barriers identified by respondents included: patient access to necessary equipment/technology (nine respondents), patient willingness to use technology (seven), bureaucratic hurdles to gaining approval for mRehab (five), mRehab is not relevant to our setting, patients or practice (five), the need for personal communication/oversight for the therapies conducted at home (four), clinician disinterest in using technology (two), lack of technical support for patients at home (two), and lack of reimbursement (one).

We also asked respondents to select their top three most important use cases for mRehab. We also gave the option of adding an “other” use case. Table 10 presents responses to six use cases listed in the questionnaire. Two use cases were cited by a large majority (69% each) of respondents: 1) supporting patient functioning at home and in the community; 2) supporting patient adherence to prescribed exercises and activities. These use cases do not necessarily involve sophisticated devices and services. More technologically advanced use cases, like real-time direct observation and communication with patients and remote biometric monitoring, were cited by less than half of the respondents.

Other use cases identified by respondents included: improving patient engagement and ownership of outcomes (4), improving access to therapy (2), medication management (1), mental health interventions (2), improving compliance especially with seating/positioning and pressure ulcer management (1), and providing data to secure or support funding for therapy.

Finally, our survey revealed an already robust rate of adoption of online therapy management platforms by survey respondents. Over 12% respondents are already using a coaching or therapy management platform. Reported uses include patient education (9%), progress tracking (7%), sending reminders, “nudges” (6%) and motivational (5%) messages, care management (4%), assistance with goal setting (3%), and voice or video communication (3%).

## 4. Discussion

The growth of technologies to support mHealth and mRehab is both a result and a cause of increasing interest by policymakers, healthcare providers, and patients themselves. Healthcare costs in the United States continue to rise at rates substantially faster than inflation (5.5% annually through 2027) [16]. This will continue to exert pressure on policymakers and providers to do more with less by realizing new efficiencies. Rising healthcare costs will be compounded by two key demographic trends: 1) the aging of the U.S. population—the “silver tsunami”—caused by aging baby boomers and longer lifespans for the population in general, and 2) the major shift in the leading causes of death for all age groups from infectious diseases and acute illnesses to chronic diseases and degenerative illnesses [17]. By 2030, the population of Americans age 65 and older will exceed the number of people 18 years and younger, growing to 20 percent of the population [18]. The shift to chronic disease management in healthcare for a rapidly growing elder population compounds existing cost pressure. Survival rates for people who experience debilitating injuries are increasing and people with disabilities are also living longer [19,20,21,22]. These trends will lead to rapidly expanding demand for technology to enhance well-being and lower healthcare costs, particularly for individuals with disabilities and chronic conditions. 

The survey data presented here indicate recognition of considerable need for mRehab technologies and broad acceptance by rehabilitation professionals. Overall, clinicians across all specialties reported that most of their patients need additional therapy after in-patient discharge or between outpatient visits. However, compared to the other professions, medical professionals reported their patients needed the least additional therapy. Physical therapists, occupational therapist, and the speech language pathologists overall indicated that their patients needed somewhat more therapy post-discharge or between outpatient visits. Recreational therapists and mental health professionals reported that their patients needing the most additional therapy. These results may reflect the duties and obligations of the several professions to the patient: medical professionals focus on stabilizing patients in the short term; physical, occupational and speech therapists are dedicated to building strength and skills over a more extended period (weeks or months); and recreation therapists and mental health professionals focus on life-long health, fitness and wellness. Longer time horizons might increase the likelihood that the intervention or therapy will benefit if also practiced in the patient home or community.

The two most critical use cases for mRehab interventions and technologies identified by respondents were: 1) supporting patient functioning at home and in the community; and 2) and patient adherence to prescribed exercises and activities. Both use cases were cited by 69% of respondents.

Survey respondents also identified substantial barriers to their ability to adopt and implement new mRehab technologies. Two key barriers are: 1) low levels of comfort integrating mRehab into practice and 2) perceived lack of knowledge of available mRehab solutions. Almost half of the respondents reported not feeling comfortable integrating mRehab technologies into their practice, and a large majority (78%) reporting not being knowledgeable about the needed technologies. Additionally, respondents identified the inability of patients to learn and correctly use mRehab technology as the top barrier to adoption and use (cited by 72%).

These responses suggest considerable uncertainty due to lack of knowledge of available technologies and insufficient training (for both clinicians and patients) in their use. Additionally, clinicians may need information and guidance to evaluate emerging technologies and understand how technology-based interventions can be incorporated into practice. This uncertainty and need for guidance will likely increase as the pace of development continue accelerate. Education and guidance for patients on proper use of prescribed technologies will also be an ongoing need, placing an additional requirement on clinicians—learning how to train patients in effective use of these innovative technologies.

The degree of support or hesitance to utilize mHealth/mRehab interventions and technologies among clinicians in rehabilitation specialties might partly be explained by how international and US-based professional organizations have addressed the opportunities and concerns. The World Health Organization (WHO) views “digital health” solutions as a key tool to strengthen national health systems in order to support the goal of Universal Health Coverage (UHC). According to the WHO: “Digital technologies provide concrete opportunities to tackle health system challenges, and thereby offer the potential to enhance the coverage and quality of health practices and services” [23].

This view is reflected in the World Federation of Occupational Therapists (WFTO), whose Statement of Position on states: “Telehealth is an appropriate delivery model for occupational therapy services when in-person services are not possible, practical, or optimal for delivering care and/or when service delivery via telehealth is mutually acceptable to the client and provider” [24]. In the United States, the main professional organization for each of the three core therapy specializations (physical, occupational and speech therapy) have published statements that support use of telehealth/mHealth [25,26,27]. Like the WHO and WFTO, these organizations emphasize the ability to extend therapy services to more patients in a more flexible way to the home and community by using of technology supported interventions, which is also the most critical use-case identified by survey respondents.

The caution identified by clinicians responding to our survey might also be reflected in the official positions of these organizations, which emphasize the need to ensure that the intervention and the technology is appropriate to patient needs, competency and context. The WFTO states the concern concisely: “… therapy services via telehealth should be appropriate to the individuals, groups and cultures served, and contextualized to the occupations and interests of clients.” The American Speech-Hearing-Language Association (ASHA) provides a detailed lists of things which need to be considered before adopting or participating in “telepractice”, including: patient privacy, ethical considerations (e.g., not exceeding the scope of the therapists qualifications), licensure, reimbursement from insurance plans for services, client selection, and environmental considerations. These types of concerns might partly explain why almost half of the survey sample reported low levels of comfort integrating mRehab technologies into their practice.

Additionally, the Technology Acceptance Model (TAM) might partly explain the degree of readiness of rehabilitation clinicians to integrate mRehab interventions into their practice. The model posits that perceived usefulness (PU) and perceived ease of use (PEOU) are the two main factors that influence whether an individual will accept new technology [28]. Each of these factors, in turn, are potentially composed of several discrete variables like demonstrability of impact, job relevance, complexity, compatibility with existing technology and processes, enjoyment, etc. Perceived utility and ease of use have been shown to affect acceptance in other studies on medical technology, including use of telemedicine in an intensive care unit (ICU) program [29]; a clinical decision support system for primary care physicians [30]; a clinical decision support system for medications in primary care [31]; and adoption of mobile and wearable technology into the practice of physiotherapists [32].

### Limitations of the Study

Limitations of this research center on the convenience sampling method, which is commonly used when sampling a population that can be difficult to reach. This sampling method requires caution in generalizing results from the sample to the larger population due to potential biases. Despite these limitations, convenience sampling can be effective for exploratory and discovery research to uncover substantive aspects of the research focus and to identify possible trends. Additionally, the present study did not investigate the role of clinician training via their professional organizations, nor the impact of perceived usefulness and ease of use on adoption of mRehab interventions and technology into clinical practice. The questionnaire constitutes only an initial investigation into clinician’s perceptions of opportunities and barriers to using mRehab interventions. As the field of mRehab and supporting technologies continue to mature, these will be critical areas of inquiry. 

## 5. Conclusions

The technologies needed to support mRehab have been maturing at a rapid pace. Meanwhile, clinicians almost universally recognize that many of their patients leave their clinic with unmet rehabilitation needs that could be met outside the clinic. Together these trends suggest accelerating adoption of mRehab technologies. However, obstacles remain. The potential benefit is recognized, but providers need additional knowledge and support to comfortably incorporate these approaches into practice, understandable given the emerging state of the field. Survey findings suggest that more systematic effort is needed to support clinicians’ successful adoption of emerging and highly promising mRehab solutions.

## Figures and Tables

**Table 1:** Respondents by profession (number and percentage of sample).

Professional Specialization	Count (*n* = 505)	Percentage (%)
Physician	13	2.6
Non-Physician Medical (Physician Asst, Nurse Practitioner, Nurse)	13	2.6
Physical Therapist	72	14.3
Occupational Therapist	104	20.6
Speech–Language Pathologist	166	32.9
Recreational Therapist	57	11.3
Mental Health (Psychologist or Counselor)	54	10.7
Other Professions	26	5.1

**Table 2:** Respondents by rehabilitation population served (number and percentage of sample).

Rehabilitation Population Served	Count (*n* = 505)	Percentage (%)
Acquired Brain Injury (ABI)	375	74.3
Neurodegenerative Disease (NDD)	300	59.4
Musculoskeletal Injury/Disorder	198	39.2
Cardiovascular Disease (CVD)	181	35.8
Cancer	178	35.2
Spinal Cord Injury (SCI)	172	34.1
Other Populations	119	23.6

**Table 3:** Clinical environments of respondents (number and percentage of sample).

Clinical Environment	Count (*n* = 505)	Percentage (%)
Inpatient acute	146	28.9
Inpatient rehab	203	40.2
Outpatient clinic	243	48.0
Skilled nursing facility	72	14.3
Home health	48	9.5
Other environments	73	14.5

**Table 4:** Percentage of patients who require additional therapeutic interventions (excluding medications) after discharge from acute inpatient rehabilitation care and between visits to outpatient program, by profession.

Professional Specialization	After Discharge: Percentage of Patients (%)	Between Outpatient Visits: Percentage of Patients (%)
All Professions	73.9	55.3
Physician	56.1	40.3
Non-Physician Medical (Physician Assistant, Nurse Practitioner, Nurse)	44.5	38.3
Physical Therapist	73.6	55.7
Occupational Therapist	70.4	48.9
Speech–Language Pathologist	75.3	57.8
Recreational Therapist	79.7	68.0
Mental Health (Psychologist or Counselor)	83.3	53.6

**Table 5:** Percentage of respondents who prescribe specific exercises and interventions for patients to work on outside of the clinic or at home, by profession.

Professional Specialization	Percentage of Respondents (%)
All Professions	71.3
Physician	53.8
Non-Physician Medical (Physician Assistant, Nurse Practitioner, Nurse)	50.0
Physical Therapist	76.4
Occupational Therapist	68.9
Speech–Language Pathologist	85.5
Recreational Therapist	42.6
Mental Health (Psychologist or Counselor)	74.1
Other Professions	48.0

**Table 6:** Level of comfort integrating mobile rehabilitation (mRehab) technologies into practice and level of knowledge of current rehabilitation technologies, by profession.

Professional Specialization	Very or Extremely Comfortable (%)	Very or Extremely Knowledgeable (%)
All Professions	50.9	22.8
Physician	66.7	36.4
Non-Physician Medical (Physician Assistant, Nurse Practitioner, Nurse)	92.3	30.8
Physical Therapist	50.0	30.6
Occupational Therapist	44.2	24.0
Speech–Language Pathologist	53.0	24.8
Recreational Therapist	44.6	9.1
Mental Health (Psychologist or Counselor)	50.0	9.3
Other Professions	53.8	30.8

**Table 7:** Level of comfort integrating mRehab technology into respondent practice, by age range (number of respondents).

Age Range	Not at All Comfortable	Not so Comfortable	Somewhat Comfortable	Very Comfortable	Extremely Comfortable
22–30	5	32	39	11	0
31–40	14	57	54	42	10
41–50	5	23	27	20	5
51+	9	26	31	8	4

**Table 8:** Perceived level of knowledge regarding current rehabilitation technology for respondent clinical specialty or patient population (number of respondents).

Age Range	Not at all Knowledgeable	Only Slightly Knowledgeable	Moderately Knowledgeable	Very Knowledgeable	Extremely Knowledgeable
22–30	5	32	39	11	0
31–40	14	57	54	42	10
41–50	5	23	27	20	5
51+	9	26	31	8	4

**Table 9:** Barriers that might limit effectiveness of mRehab effectiveness in supporting between-visit therapy interventions, all professions (select top three).

Potential Barriers	Percentage of Respondents (%)
Patient unable to learn or correctly use the technology	71.5
Patients with limited or no access to internet services	63.4
Cost vs. reimbursement (verifiable return on investment)	32.3
Patient concern for security and privacy	25.5
Hassle and time commitment for clinicians to adapt (learn, etc.)	24.2
Concerns over accuracy and reliability of technology	22.0
Concerns over liability and licensing	19.8
Improvement in patient outcomes or clinical efficiency may not be sufficient to justify change in practice	12.3

**Table 10:** Most critical use case for mRehab, all professions, all professions (select top three).

Use Cases	Percentage of Respondents (%)
Support patient functioning at home and in the community	68.9
Support patient adherence to prescribed exercises and activities	68.7
Enable real-time, direct observation/communication with patient	47.9
Enable remote biometric monitoring of patients’ activity	33.5
Enable patients’ self-reporting of outcomes data	31.3
Enable remote environmental monitoring of activity at home	17.4
I do not believe mRehab can support therapy for my patients	2.0

## Supplementary Materials

The following are available online at https://www.mdpi.com/1660-4601/16/21/4220/s1, File S1: Questionnaire.
