# Supplementary material for: Clinician Perspectives on mRehab Interventions and Technologies for People with Disabilities in the United States: A National Survey
Source: Int J Environ Res Public Health. 2019 Oct 31;16(21):4220. doi: 10.3390/ijerph16214220 (PMC6862627; doi:10.3390/ijerph16214220)
Supplement: Supplementary file 1 [file ijerph-16-04220-s001.pdf]

Welcome!

Introduction

Thank you for participating in our survey. This short questionnaire is intended as a first step in identifying clinicians' perceptions of the needs and barriers to adoption of technology-based rehabilitation interventions inside and outside the clinic for people with disabilities and chronic conditions. In turn, the information collected from this survey will be used to help researchers, designers, and engineers create new mRehab solutions to meet the needs of people with disabilities and chronic conditions.

We use the term "mRehab" to refer to: *interventions that involve delivery of rehabilitation services and collection of health-related information using mobile telecommunication devices – including cell phones, tablet computers, "wearable" tracking or sensor-based recording devices, and the Internet.*

Our definition of mRehab deliberately excludes traditional telemedicine and telehealth which typically relies on direct one-to-one or one-to-many intervention by a clinician with patients via voice and video communication.

Your answers are important, even if you do not currently utilize mRehab interventions and technologies in your practice. The survey takes about 8-10 minutes to complete.

All participants will receive a $5.00 Starbucks Coffee gift card -- a different kind of mRehab therapy! Just be sure to include your name, email address, year of birth, and zip code at the end of this questionnaire.

We also hope you will consider joining our mRehabTech Network, our national network of clinicians, researchers and engineers working with information and communication technologies to promote rehabilitation beyond the clinic. If interested, at the end of this questionnaire you can select "Yes" to join.

ABOUT US
This survey is being conducted by the Rehabilitation Engineering Research Center for Community Living, Health and Function (LiveWell RERC), funded by a 5-year grant from the National Institute on Disability, Independent Living and Rehabilitation Research in the U.S. Department of Health and Human Services (grant number 90RE5023). For more information, please visit us at: www.livewellrerc.org or contact project director John Morris at john.morris@shepherd.org, 404-295-7178.

This research project has been reviewed and approved by the Research Review Committee at Shepherd Center to ensure the protection of participants.

If you have questions or concerns about this research, please contact:
Ismari Clesson at 404-350-7651; ismari_clesson@shepherd.org

1. I agree to participate in this survey on mRehab interventions and technologies.

- ◯ Yes
- ◯ No

LiveWell RERC *for* ICT ACCESS

Clinician perspectives on mRehab interventions and technologies v.10

Part 1: About you

Please answer the following questions to let us know more about you.

2. What is your profession?

- ◯ Physician
- ◯ Physician assistant
- ◯ Nurse or nurse practitioner
- ◯ Physical therapist
- ◯ Occupational therapist
- ◯ Speech language pathologist
- ◯ Recreational therapist
- ◯ Counselor or social worker
- ◯ Psychologist
- ◯ Case manager or care coordinator
- ◯ Other (please specify)

3. How many years have you worked in this profession?

4. Which patient population do you CURRENTLY provide services to? (check all that apply)

- [ ] Acquired brain injury (ABI), including traumatic and non-traumatic injuries, such as stroke or anoxic brain injury
- [ ] Spinal cord injury (SCI)
- [ ] Neurodegenerative disease (e.g.,MS, Parkinson's disease, ALS)
- [ ] Cardiovascular disorder
- [ ] Musculoskeletal injury or disorder
- [ ] Cancer
- [ ] Other (please specify)

5. What type of clinical unit(s) do you work in? (check all that apply)

- [ ] Inpatient acute care
- [ ] Inpatient rehab
- [ ] Outpatient clinic
- [ ] Skilled nursing facility (SNF)
- [ ] Home health
- [ ] Other (please specify)

6. Do you personally use any wearable tracking or sensor-based devices, like a smartwatch, tracking bracelet (Fitbit, etc.), pendant or clip-on tracker?

- ( ) Yes
- ( ) No

If yes, please specify what device/s you have for your personal use.

LiveWell RERC for ICT ACCESS

Clinician perspectives on mRehab interventions and technologies v.10

Part 2: About your patients' post-acute and between-visits care needs

Please answer the following questions to help us identify the clinical perceptions of

the needs and barriers to implementing rehabilitation interventions post acute and between-visits care.

7. Approximately what percentage of YOUR PATIENTS generally need additional therapeutic interventions (excluding medications) AFTER DISCHARGE from acute care?

8. Approximately what percentage of YOUR PATIENTS generally need additional therapeutic interventions (excluding medications) BETWEEN VISITS to the outpatient/day program?

9. How much ADDITIONAL THERAPEUTIC INTERVENTION beyond clinical visits is needed for YOUR PATIENTS to reach their rehabilitation goals in the following areas?

	None	A little	Some	Quite a bit	Substantial	N/A
Stength	○	○	○	○	○	○
Flexibility	○	○	○	○	○	○
Balance	○	○	○	○	○	○
Range of motion	○	○	○	○	○	○
Endurance	○	○	○	○	○	○
Breathing	○	○	○	○	○	○
Pain management	○	○	○	○	○	○
Tone/spasticity management	○	○	○	○	○	○

10. How much ADDITIONAL TRAINING beyond clinical visits is needed for YOUR PATIENTS to reach their rehabilitation goals in the following areas?

	None	A little	Some	Quite a bit	Substantial	N/A
Physical self-care (e.g., skin, bowel and bladder)	○	○	○	○	○	○
Cognitive self-care (e.g., stress management, emotional regulation)	○	○	○	○	○	○
Safe mobility (e.g., transfers, wheelchair skills)	○	○	○	○	○	○
Upper extremity function	○	○	○	○	○	○
Activities of daily living (e.g., bathing, dressing, cleaning, cooking)	○	○	○	○	○	○
Sleep management	○	○	○	○	○	○
Medication management	○	○	○	○	○	○

11. How much ADDITIONAL COGNITIVE SUPPORT beyond clinical visits is needed for YOUR PATIENTS to meet their rehabilitation goals in the following areas?

	None	A little	Some	Quite a bit	Substantial	N/A
Vision or visual processing	○	○	○	○	○	○
Hearing or auditory processing	○	○	○	○	○	○
Mood	○	○	○	○	○	○
Reasoning skills	○	○	○	○	○	○
Memory	○	○	○	○	○	○
Attention	○	○	○	○	○	○
Planning skills	○	○	○	○	○	○
Impulse control	○	○	○	○	○	○
Communication skills	○	○	○	○	○	○
Problem solving	○	○	○	○	○	○

12. Are there specific exercises and interventions that you prescribe for YOUR PATIENTS to work on outside of the clinic or at home?

- ◯ Yes
- ◯ No

If yes, please specify which exercises or interventions.

Clinician perspectives on mRehab interventions and technologies v.10

Part 3. About the use of mRehab technology

The previous section asked about your patients' needs in general. This section focuses more specifically on the use of mRehab technology in your practice for your patients.

Definition: mRehab" refers to interventions that involve delivery of rehabilitation services and collection of health-related information using mobile telecommunication devices – including cell phones, tablet computers, “wearable” tracking or sensor-based recording devices, and the Internet.

13. Do you think that mobile or internet technology could be effective in supporting post-acute or between-visit (outside of the clinic) therapy interventions for YOUR PATIENTS?

- ◯ Yes
- ◯ No

14. How comfortable would you be with integrating mRehab technology into YOUR PRACTICE?

- ◯ Extremely comfortable
- ◯ Very comfortable
- ◯ Somewhat comfortable
- ◯ Not so comfortable
- ◯ Not at all comfortable

15. Would you be open to using mobile and internet technology for: 1) initial evaluations, and/or 2) follow-up appointments with YOUR PATIENTS?

- ◯ Yes, for both (initial and follow-up appointments)
- ◯ Yes, for initial evaluation only
- ◯ Yes, follow-up appointments only
- ◯ No
- ◯ Other (please specify)

* 16. What BARRIERS might limit or detract from mobile and internet technology's effectiveness in supporting post-acute and between-visits therapy interventions? (please select top 3)

- ☐ Patients unable to learn and/or correctly use the technology
- ☐ Patients with limited or no access to internet services
- ☐ Hassle and time commitment demanded from clinicians to adopt (learn, train, integrate, etc.)
- ☐ Improvement in patient outcomes or clinical efficiency is not significant enough to change practices
- ☐ Concerns over accuracy and reliability of system
- ☐ Patient concern over security and privacy
- ☐ Clinician concern over liability and licensing
- ☐ Cost vs. reimbursement (verifiable return on investment)
- ☐ Other (please specify)

* 17. What do you believe are the MOST CRITICAL USE CASES for mobile or internet technology support in post-acute or between-visits therapy interventions (please select top 3)

- ☐ Support patient adherence to prescribed exercises or activities
- ☐ Support patient functioning at home and in the community
- ☐ Enable real-time, direct observation or communication between you and your patients
- ☐ Enable patients' self-reporting of outcomes data via forms and/or questionnaires
- ☐ Enable remote biometric monitoring of your patients' activity using mobile apps and/or wearable devices (e.g. step counting, heart rate tracking, sleep data reproting)
- ☐ Enable remote environmental monitoring using sensors in patients/ homes
- ☐ I don't believe mobile or internet technology can significantly support therapeutic interventions for my patients outside of the clinic
- ☐ Other (please specify)

Part 4. Online health coaching platforms

Online health coaching platforms have emerged to provide multiple components of clinical support to patients at home and in the community. These features often include goal setting, progress tracking, motivational messaging, reminders and nudging, patient education, care management, direct voice and video communication, among other features.

18. Are you currently using any ONLINE COACHING PLATFORMS to support your patients' care?

- ◯ Yes
- ◯ No

19. If currently using an ONLINE COACHING PLATFORM to support patient care, what do you primarily use it for?

- ☐ Goal setting
- ☐ Progress tracking
- ☐ Motivational messaging
- ☐ Reminders and nudging
- ☐ Patient education and/or training
- ☐ Care management
- ☐ Direct voice and video communication
- ☐ Other (please specify)

Clinician perspectives on mRehab interventions and technologies v.10

Part 5. Learning about new rehabilitation technology solutions

20. How knowledgeable do you feel regarding current rehabilitation technology for your clinical specialty or patient population?

- ◯ Extremely knowledgable
- ◯ Very knowledgable
- ◯ Moderately knowledgeable
- ◯ Only slightly knowledgeable
- ◯ Not at all knowledgeable

21. What sources of information do you rely on to learn about advances in rehabilitation technology? (Check all that apply.)

- ☐ Mainstream news (television, radio, newspapers, magazines)
- ☐ Online information sources (blogs, websites focusing on rehabilitation medicine or technology)
- ☐ Professional conferences (AOTA, APTA, ASHA, ACRM, RESNA, etc.)
- ☐ Professional or research journals
- ☐ In-service presentations given by researchers and/or engineers
- ☐ Directly from technology vendors
- ☐ Directly from your patients and their families
- ☐ Other rehabilitation clinicians
- ☐ Other (please specify)

22. Please provide any additional comments or observations regarding mRehab interventions and technology.

LiveWell RERC for ICT ACCESS

Clinician perspectives on mRehab interventions and technologies v.10

Thank you!

Here you can enter your email address so we can send your$5.00 Starbucks Coffee gift card.

We also invite you to join mRehabTech Network, our national network of rehabilitation clinicians, researchers and engineers. If you click "yes", you may withdraw at any time. This is purely a

research and community-building endeavor. Your information will not be shared outside of this network -- as we promised in our research protocol approved by Shepherd Center's Research Review Committee.

23. To receive your $5.00 Starbucks gift card, please fill in the following fields. We are asking for the minimum amount of information while still preserving your privacy. We are bound by our commitment to human subjects research protections, and will not share any identifiable information outside of project staff at Shepherd Center.

Name ____________________

Year of birth ____________________

ZIP/Postal code __________

Email address for your gift card ____________________

24. Please indicate if you would like to join our mRehabTech Network, our national network of clinicians, researchers and engineers working with information and communication technologies to promote rehabilitation beyond the clinic.

- ○ Yes
- ○ No
